# The role of diabetes mellitus and hypertension in chronic kidney disease

**DOI:** 10.12861/jrip.2014.31

**Published:** 2014-12-01

**Authors:** Seyed Bahman Ghaderian, Seyed Seifollah Beladi-Mousavi

**Affiliations:** ^1^Department of Internal Medicine, Faculty of Medicine, Ahvaz University of Medical Sciences, Ahvaz, Iran; ^2^Chronic Renal Failure Research Center, Department of Internal Medicine, Ahvaz University of Medical Sciences, Ahvaz, Iran

**Keywords:** Chronic renal failure, Diabetes mellitus, Hypertension

**Implication for health policy/practice/research/medical education:**

Everyone with risk factors of chronic kidney disease (CKD) such as high blood pressure, diabetes mellitus, metabolic syndrome, family history of CKD and proteinuria should be educated about the benefits of early identification of the disease and subsequent kidney protection through appropriate interventions.

With interest, we read the article by Hernandez *et al*. about increasing awareness of chronic kidney disease and aging (1). We agree with the authors of the article that, the diabetes mellitus and hypertension are the leading causes of chronic kidney disease (CKD).

Over the last decade, different kinds of glomerulonephritis (GN) were the leading causes of CKD in the world, too. However, possibly due to lifestyle changes and increasing prevalence of obesity, diabetes and hypertension and because of more available aggressive treatment of GN, it is well established that diabetes and hypertension are now the primary causes of CKD in developed countries (2-6). However, it seems that there is an important difference between causes of end-stage renal disease (ESRD) in developed and developing countries. In contrast to the United States and other developed countries, in developing countries, the cause of ESRD among significant percent of patients is the unknown etiology possibly due to late presentation and late referral of patients with CKD to the specialists (7-12). For example, in a report from Iran, Beladi-Mousavi *et al*. evaluated the cause of CKD among 1000 adult ESRD patients from January 1999 to March 2010. Although according to the result of this study, diabetic nephropathy and hypertensive nephrosclerosis were the most common causes of ESRD, however in the significant number of the patients (n=242, 24.2%), the causes of ESRD were unknown (8). The results of other studies which have done in Iran, for example Haghighi *et al*. in 2002 (7), Malekmakan *et al*. in 2009 (11) and Salahi *et al*. in 2004 (12) were also similar.

In some developing countries like Iran, the significant percent of patients with CKD are presented to the nephrologist with the severe symptoms of uremia and late stage of the disease. Unfortunately at this time, determining the primary causes of CKD is not possible.

Renal biopsy is also not helpful and it cannot determine the cause of CKD at the end-stage of the disease. Regardless of the causes of CKD, histologic findings of kidney biopsy at the late stage of CKD are glomerulosclerosis, tubular atrophy and interstitial fibrosis and therefore renal biopsy is not helpful. In addition, in most of the patients with CKD, the sizes of kidneys are gradually decreased and kidney biopsy is not possible at the end stage. It is associated with increment risk and therefore kidney biopsy not recommended (13,14).

In conclusion, although the diabetic nephropathy and hypertensive nephrosclerosis are also the most common causes of ESRD in developing countries, however, possibly because of unawareness of patients with CKD and late referral of patients with CKD to the nephrologists, the causes of ESRD in the significant percent of patients in developing countries are still unknown and therefore everyone with risk factors of CKD such as high blood pressure, diabetes mellitus, metabolic syndrome, family history of CKD and proteinuria have to be educated about the benefits of early identification of the disease and subsequent kidney protection through appropriate interventions.

## Author’s contributions

All authors contributed to the paper equally.

## Ethical considerations

Ethical issues (including plagiarism, informed consent, misconduct, double publication and redundancy) have been completely observed by authors.

## Conflict of interests

The authors declared no competing interests.

## Funding/Support

None.
